# Facile Fabrication of a PDMS@Stearic Acid-Kaolin Coating on Lignocellulose Composites with Superhydrophobicity and Flame Retardancy

**DOI:** 10.3390/ma11050727

**Published:** 2018-05-03

**Authors:** Zhe Wang, Xiaoping Shen, Temeng Qian, Junjie Wang, Qingfeng Sun, Chunde Jin

**Affiliations:** 1 School of Engineering, Zhejiang A&F University, Hangzhou 311300, China; donjade@163.com (Z.W.); sxp1031@hotmail.com (X.S.); teemeeng@163.com (T.Q.); jjwang7475@163.com (J.W.); 2 Key Laboratory of Wood Science and Technology, Hangzhou 311300, China

**Keywords:** lignocellulose composites, kaolin, PDMS, superhydrophobic, flame retardancy

## Abstract

The disadvantages such as swelling after absorbing water and flammability restrict the widespread applications of lignocellulose composites (LC). Herein, a facile and effective method to fabricate superhydrophobic surfaces with flame retardancy on LC has been investigated by coating polydimethylsiloxane (PDMS) and stearic acid (STA) modified kaolin (KL) particles. The as-prepared coatings on the LC exhibited a good repellency to water (a contact angle = 156°). Owing to the excellent flame retardancy of kaolin particles, the LC coated with PDMS@STA-KL displayed a good flame retardancy during limiting oxygen index and cone calorimeter tests. After the coating treatment, the limiting oxygen index value of the LC increased to 41.0. Cone calorimetry results indicated that the ignition time of the LC coated with PDMS@STA-KL increased by 40 s compared with that of uncoated LC. Moreover, the peak heat release rate (PHRR) and the total heat release (THR) of LC coated with PDMS@STA-KL reduced by 18.7% and 19.2% compared with those of uncoated LC, respectively. This LC coating with improved water repellency and flame retardancy can be considered as a potential alternative to protect the lignocellulose composite.

## 1. Introduction

Lignocellulose composites (LC) are widely popular as the furniture and interior decoration materials in our daily life, which is attributed to their low cost, relatively high mechanical properties and easy machinability [1,2]. However, LC are hydrophilic and hygroscopic materials due to the existence of the hydrophilic hydroxyl groups from cellulose and hemicelluloses of lignocellulose fibers [3]. After absorbing water, lignocellulose composites exhibit rapid swelling, resulting in the decrease of their mechanical properties and the reduction of their service life. Therefore, hydrophobization treatments are exceedingly important for the hydrophilic lignocellulose composites to improve their durability.

Inspired by natural lotus leaves, superhydrophobic coatings have gained tremendous research interest for improving hydrophobicity of wood-based materials [4,5]. It is well known that the formation of superhydrophobic surfaces is attributed to the dual function of micro/nano- hierarchical roughness and low surface energy [6]. Up to now, a variety of methods have been developed to fabricate superhydrophobic surfaces on wood-based materials, such as hydrothermal treatment [7,8], sol-gel method [9,10], dip or spray-coating [11,12,13,14], soft lithography [15,16], “paint + adhesive” method [17,18], and so on. Among “paint + adhesive” methods, PDMS as adhesive has been successfully used to fabricate various robust and durable superhydrophobic surfaces [19,20,21]. However, there are few studies on the fabrication of lignocellulose composites superhydrophobic coatings.

In addition, lignocellulose composites are flammable due to their organic nature, which limits their applications in indoor environments [22]. In order to be used safely, the flame retardant properties of lignocellulose composites should be taken into consideration in high levels. Adding halogen-containing flame retardants is a conventional method to improve flame retardant properties of lignocellulose composites. However, the leakage of hazardous flame retardants could have a negative effect on the environment and human health [23]. In recent years, halogen-containing compounds have been gradually replaced by nitrogen- or phosphorus-containing flame retardants [24,25]. Nevertheless, phosphorus-containing flame retardants (aluminum hypophosphite) also pose a fire risk. It will decompose and release phosphine which is spontaneously flammable in the air [26]. Consequently, there is an urgent need to improve flame retardant properties of lignocellulose composites by more environmentally friendly and efficient methods. Kaolin mainly consists of aluminosilicate with the formula: Al_2_Si_2_O_5_(OH)_4_ [27]. Kaolin has been widely used in the pigment or surface coating, as well as in the reinforcement and flame retardant of polymers due to its high performance and low cost [27,28,29,30]. Furthermore, there are few studies on the preparation of superhydrophobic surfaces using kaolin particles [31,32]. However, to the best of our knowledge, there are no data on the fabrication of the multifunctional lignocellulose composites surfaces with superhydrophobicity and flame retardancy using kaolin particles.

In this work, we reported a facile process to fabricate multifunctional superhydrophobic coatings on lignocellulose composites by the “paint + adhesive” method. After being modified by STA, KL particles transformed into superhydrophobic STA-KL. The STA-KL particles endowed the coating with roughness and flame retardancy and PDMS provided enhanced robustness as an adhesive. This paper asserts that the obtained superhydrophobic and flame retardant surfaces could broaden the application fields of lignocellulose composites in real life.

## 2. Materials and Methods

### 2.1. Materials

Lignocellulose fibers (20–100 mesh), consisting of a blend of softwood and hardwood fibers from different species, were provided by Great World Group (Ningbo, China). The lignocellulose fibers were produced through the fiber hot grinding process. Chitosan was purchased from Macklin Biochemical Co., Ltd. (Shanghai, China). Glutaraldehyde (25 wt. %), absolute ethanol and stearic acid (STA) were provided by Sinopharm Chemical Reagent Co., Ltd. (Shanghai, China). Kaolin particles (<2.5 μm) were purchased from Aladdin (Shanghai, China). Polydimethylsiloxane (PDMS) and its curing agent were supplied by Dow Corning Company (Midland, MI, USA).

### 2.2. Preparation of Lignocellulose Composites

Crosslinking chitosan as the adhesive of lignocellulose composites was fabricated according to the previous studies [33]. Firstly, chitosan was solubilized in an acetic acid solution of 1.5% (*w*/*v*) to obtain a 2% (*w*/*v*) chitosan mixture at room temperature. After the formation of chitosan mixture, 20 wt. % (*w*/*w*, to chitosan) glutaraldehyde was dropwise injected into the chitosan mixture under stirring. After the formation of chitosan hydrogel, the lignocellulose fibers were mixed with chitosan hydrogel following a mass ratio of 5.0:100 (chitosan to lignocellulose fibers). Finally, the blended fibers were hot pressed at 180 °C, 4.5 MPa to form a board with a size of 200 mm × 200 mm × 8 mm.

### 2.3. Preparation of Stearic Acid Modified Kaolin (STA-KL) Powders

Firstly, 0.3 g of stearic acid was solubilized in 15 mL of absolute ethanol solution in a round-bottom flask with magnetic stirring for 30 min. After that, 4.0 g of kaolin particles were added to the stearic acid solution, and sonicated for 30 min. Then, the mixture was heated at 100 °C for 2 h by an oil bath under magnetic stirring to obtain STA modified KL suspension. Afterwards, the STA-KL particles were obtained from the suspension by filtering. Finally, the STA-KL particles were dried at 120 °C for 2 h.

### 2.4. Preparation of PDMS@STA-KL Coating on Lignocellulose Composites

**Figure 1:**
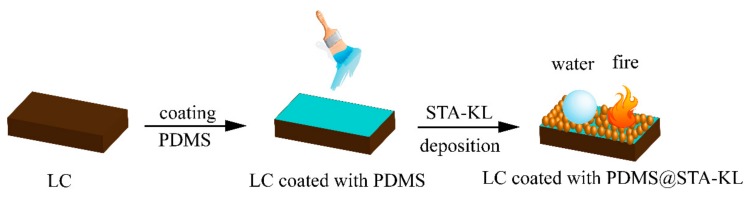
Schematic illustration of the procedure to fabricate Polydimethylsiloxane@stearic acid-kaolin (PDMS@STA-KL) coating on Lignocellulose composites (LC) surface.

The fabrication process of the PDMS@STA-KL coating is illustrated in Figure 1. In a typical fabricating process, 5.0 g of PDMS and 0.5 g of curing agent were mixed at room temperature. After that, the PDMS blended with curing agent were coated on lignocellulose composites using a brush. Then, the lignocellulose composites coated with PDMS were further covered by the as-prepared STA-KL particles. Finally, a superhydrophobic coating with flame retardancy on the lignocellulose composites was obtained after drying at 70 °C.

### 2.5. Characterization

Scanning electron microscopy images of lignocellulose composite surface before and after coating treatment were observed by SU8010 (Hitachi, Tokyo, Japan). The surface chemical compositions of the unmodified and modified KL particles were analyzed by X-ray photoelectron spectroscopy (XPS, Thermo ESCALAB 250XI, Waltham, MA, USA). The chemical group changes of the modified KL and the coated lignocellulose composites were determined by Fourier transform infrared spectroscopy (FTIR, iS10, Nicolet, Waltham, MA, USA) using the KBr pellet method. The crystalline structures of KL particles before and after modification and coated lignocellulose composite surface were characterized by X-ray diffraction (XRD, XRD-6000, Shimadzu, Kyoto, Japan). Surface wettabilities of lignocellulose composites before and after coating treatment were characterized using OCA100 contact angle test system (DataPhysics, Stuttgart, Germany) at room temperature by the average values of five measurements at different positions with water drop volume of 4 µL. Limiting oxygen index values of uncoated and coated lignocellulose composites (150 mm × 10 mm × 3 mm) were obtained by a JF-5 oxygen index instrument. Combustion parameters of uncoated and coated lignocellulose composites (100 × 100 × 8 mm^3^) were obtained using a cone calorimeter (FTT Company, Derby, UK) at an irradiance of 50 kW m^−2^.

## 3. Results and Discussion

### 3.1. XPS and FTIR Analysis

**Figure 2:**
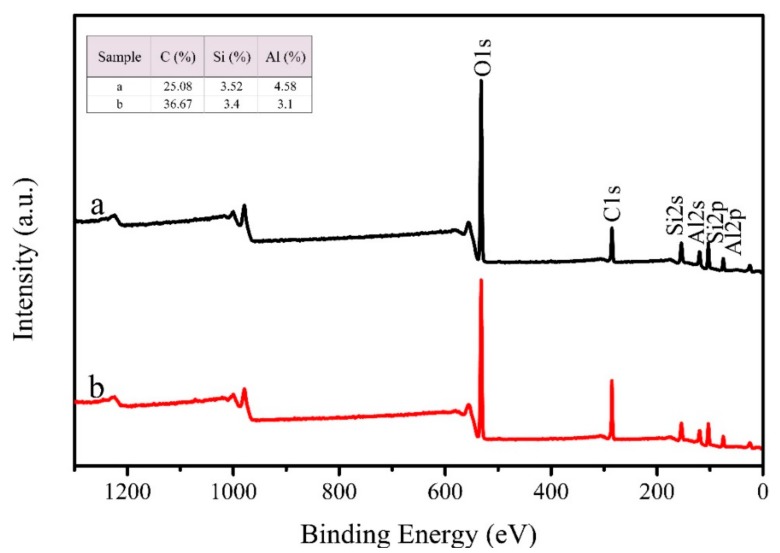
X-ray photoelectron spectroscopy (XPS) spectra of (**a**) unmodified kaolin (KL); (**b**) stearic acid (STA) modified KL.

The XPS survey spectra of KL and STA-KL are shown in Figure 2. As can be seen from Figure 2, KL and STA-KL mainly displayed six peaks at 74 eV, 103 eV, 119 eV, 154eV, 284 eV and 532 eV belonging to Al2p, Si2p, Al2s, Si2s, C1s and O1s, respectively. Compared with KL, the C1s relative intensity of STA modified KL increased markedly (from 25.08 to 36.67), which may be the reason that STA successfully covered on KL particles.

**Figure 3:**
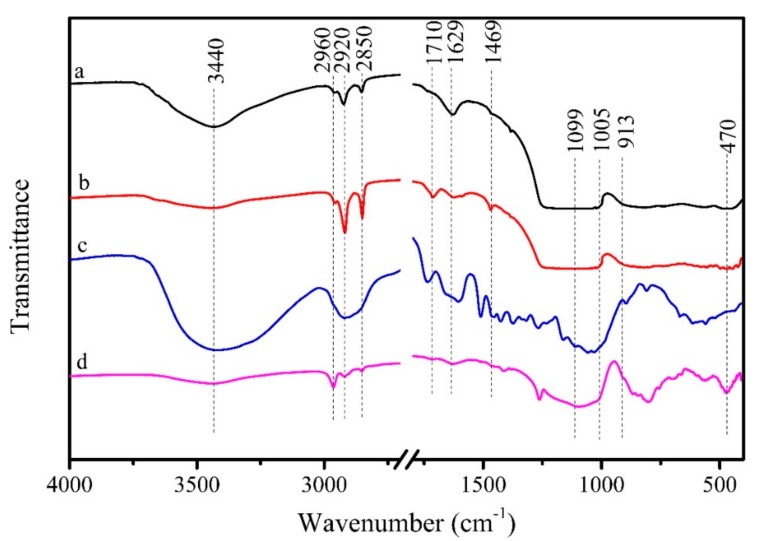
FTIR spectra of (**a**) KL; (**b**) STA modified KL; (**c**) LC; (**d**) LC coated with PDMS@STA-KL.

Figure 3 shows the FTIR spectra of KL, STA-KL, LC and LC coated with PDMS@STA-KL. As shown in Figure 3a, it can be observed that KL particles displayed two absorption peaks at 3440 and 1629 cm^−1^, which corresponded to the hydroxyl vibrations [34]. After modified by STA, the relative intensities of the above two absorption bands decreased (Figure 3b). Furthermore, there was a remarkable increase in the relative intensities of the absorption peaks at 2920 and 2850 cm^−1^, corresponding to the asymmetric and the symmetric –CH_2_ [35]. Meanwhile, STA-KL exhibited two additional absorption peaks at 1710 and 1469 cm^−1^, which were assigned to the stretching of –COO and the bending of –CH_2_ [32], respectively. These results suggested that the KL particles were successfully modified by STA. The modification process of KL particles may be the interactions between the carboxyl groups of STA and the hydroxyl groups of KL. As can be seen from Figure 3c,d, some absorption bands of LC cannot be observed after coating PDMS@STA-KL. Meanwhile, several new absorption bands were observed at 2960 cm^−1^, 2850 cm^−1^, 1710 cm^−1^, 1005 cm^−1^, 913 cm^−1^ and 470 cm^−1^ assigned to asymmetric –CH_3_, symmetric –CH_2_, –COO stretching, (Si, Al)–O asymmetric stretching, Al-OH and (Si, Al) –O bending from the STA-KL particles, respectively [36,37]. In addition, the absorption band at 1099 cm^−1^ (Si–O–Si stretching) was also observed from PDMS [4,16]. These results demonstrated that the PDMS@STA-KL coating was successfully covered on the LC.

### 3.2. XRD Analysis

**Figure 4:**
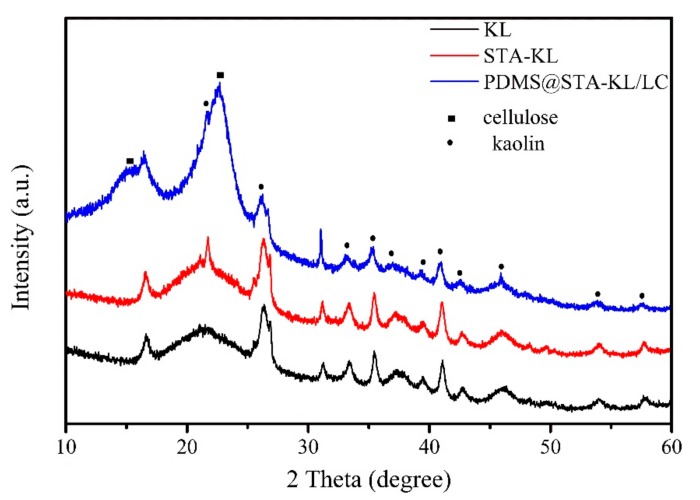
X-ray diffraction patterns of KL, STA-KL and LC coated with PDMS@STA-KL.

Figure 4 shows the crystalline structure of KL, STA-KL and LC coated with PDMS@STA-KL. As can be seen from Figure 4, after modified by STA, the crystalline structure of KL was almost unchanged, indicating that the modification had no effect on the crystalline structure of KL. This may indicate that the interactions between STA and KL did not affect its crystalline structure. The LC coated with PDMS@STA-KL displayed two typical cellulose diffraction peaks at 2θ = 15.5° and 22.7°, indicating that the coating treatment had no effect on crystalline structure of LC. Moreover, after the coating treatment, some diffraction peaks appeared at around 2θ = 21.2°, 26.4°, 32.5°, 35.4°, 37.7°, 39.3°, 41.1°, 42.7°, 45.9°, 54.3° and 57.8° assigned to the diffraction of the (−1–11), (111), (022), (−1–31), (003), (131), (−132), (041), (2–21), (−150), (−152) plane and were consistent with kaolinite-1A (PDF# 14-0164). This result further demonstrated that the STA-KL was successfully coated on the surface of LC.

### 3.3. Wettability and Surface Morphology Analysis

**Figure 5:**
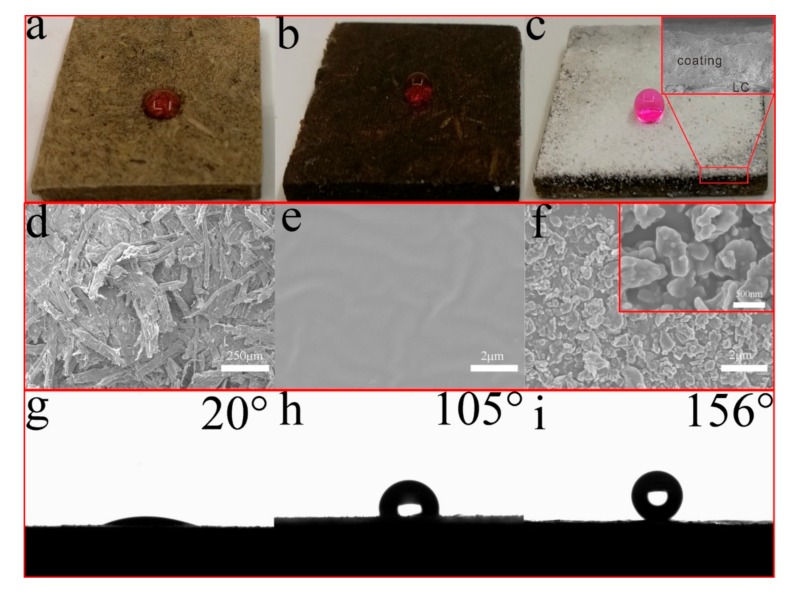
Digital photos, scanning electron microscopy (SEM) images and contact angles images of different surfaces: (**a**,**d**,**g**) LC; (**b**,**e**,**h**) LC coated with PDMS; (**c**,**f**,**i**) LC coated with PDMS@STA-KL.

**Figure 6:**
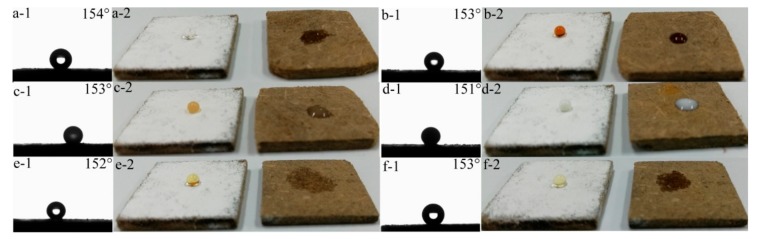
Images of different liquids on the surface of LC and LC coated with PDMS@STA-KL: (**a-1,a-2**) tea; (**b-1,b-2**) soy sauce; (**c-1,c-2**) coffee; (**d-1,d-2**) milk; (**e-1,e-2**) millet wine; (**f-1,f-2**) juice.

Figure 5a–c,g–i shows the contact angle images of water droplets on the uncoated and coated lignocellulose composites. The water droplet was immediately absorbed by the uncoated lignocellulose composites due to their hydrophilic properties (Figure 5a,g). The LC coated with PDMS showed an increased WCA (105°) (Figure 5b,h), which can be attributed to the hydrophobic property of PDMS. In contrast, it can be observed that the WCA of LC displayed a prominent increase (156°) after a dual treatment with PDMS and STA-KL particles (Figure 5c,i). As shown in Figure 5c, the water droplet stood on the coated LC with a perfect spheroidal, indicating the superhydrophobic properties of PDMS@STA-KL coating. The surface morphological changes of LC before and after the coating treatment are shown in Figure 5d–f. The LC displayed a rough structure due to the irregular arrangement of lignocellulose fibers (Figure 5d). After coating PDMS treatment, a smooth coating was observed instead of the rough structure of lignocellulose fibers (Figure 5e), indicating that the PDMS uniformly covered on the LC surface. After the further deposition, the irregular STA-KL particles were observed, which led to the formation of hierarchical structures on the LC (Figure 5f and insert). Based on Cassie and Baxter’s law, the hydrophobicity of solid materials surfaces can be improved by increasing the area taken up by air between solid and aqueous interface [38]. Therefore, this hierarchical structure creates the conditions for the formation of superhydrophobicity. Furthermore, contact angles of some domestic liquid contamination sources on the LC coated with PDMS@STA-KL were also investigated, such as tea, soy sauce, coffee, milk, millet wine and juice (Figure 6). As can be seen from Figure 6, the contact angles of all the liquid droplets on the LC coated with PDMS@STA-KL were more than 150°, which indicated that this coating had a good domestic liquids repellency.

### 3.4. Flame Retardancy Analysis

**Figure 7:**
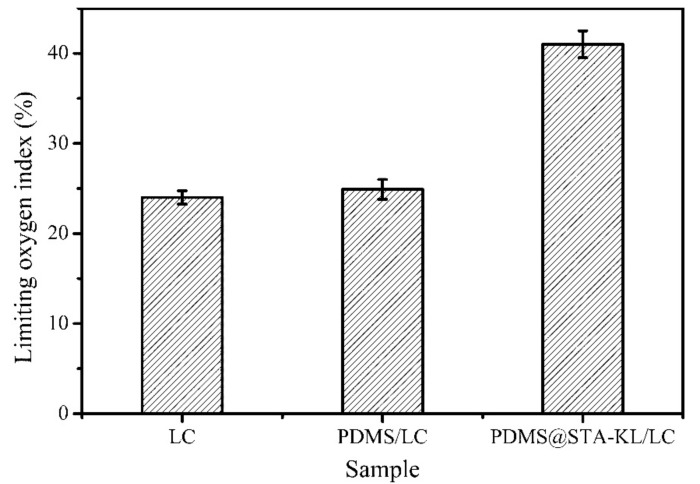
Limiting oxygen index values of LC, LC coated with PDMS and LC coated with PDMS@STA-KL.

The LOI test was carried out on the LC coated with PDMS@STA-KL to evaluate its flame retardancy. As shown in Figure 7, the LC showed a LOI value of 24.0, indicating that the LC was inflammable. Meanwhile, the LOI value of LC coated with PDMS was similar with that of LC. However, after depositing PDMS@STA-KL coating, the LOI value of the coated LC reached to 41.0 due to the flame retardancy of kaolin particles. The LOI value of LC coated with PDMS@STA-KL increased by 70.8% compared with that of LC.

**Figure 8:**
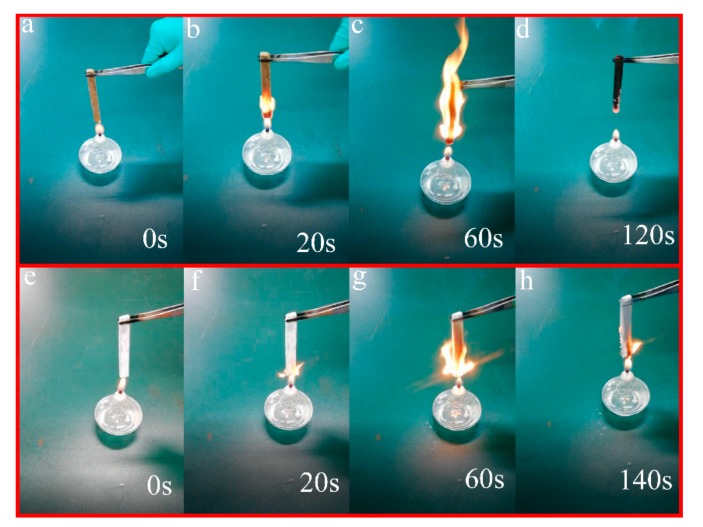
Combustion process of (**a**–**d**) LC; (**e**–**h**) LC coated with PDMS@STA-KL.

The combustion test of LC and LC coated with PDMS@STA-KL were carried out to evaluate their flame retardancy (Figure 8). As shown in Figure 8b, about one third area of LC was ignited after 20 s. However, LC coated with PDMS@STA-KL was just ignited after 20 s (Figure 8f). The whole LC was surrounded by a big fire after 60 s (Figure 8c). After coating PDMS@STA-KL, the fire spread more slowly and just burned at the bottom after 60 s (Figure 8g). These further indicated that the PDMS@STA-KL coating improved the flame retardancy of the LC.

**Figure 9:**
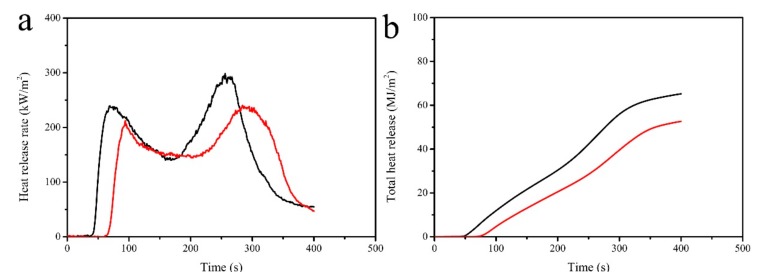
Cone calorimeter parameters of LC and LC coated with PDMS@STA-KL: (**a**) heat release rate; (**b**) total heat release.

The cone calorimeter test was used to further evaluate the flame retardancy of LC coated with PDMS@STA-KL. Figure 9 and Table 1 show the cone calorimeter test data of LC and LC coated with PDMS@STA-KL. The ignition time increased from 33 s (LC) to 73 s (LC coated with PDMS@STA-KL). This further indicated that it was more difficult to ignite the LC coated with PDMS@STA-KL. As shown in Figure 9a and Table 1, the peak heat release rate (PHRR) of the LC coated with PDMS@STA-KL (at 284 s) decreased by 18.7% compared with that of the LC (at 256 s). The fire growth rate (FIGRA) of the LC was 1.2 kW/m^2^ s, while it decreased to 0.8 kW/m^2^ s (LC coated with PDMS@STA-KL). This indicated that PDMS@STA-KL coating resulted in a slower fire growth speed and lower risk of fire hazard. Furthermore, as can be seen from Figure 9b, the total heat release of the LC coated with PDMS@STA-KL reduced by 19.2% compared with that of the LC. These confirmed that the flame retardancy of the LC was improved after coating PDMS@STA-KL.

## 4. Conclusions

In summary, a superhydrophobic surface with flame retardancy was successfully fabricated on lignocellulose composites by coating PDMS@STA-KL. This coating exhibited excellent repellency toward water (contact angle = 156°). The PDMS provided the adhesion between LC and STA-KL and the STA-KL particles contributed to the rough morphology of the coating. Besides, the LOI values of lignocellulose composites increased by 70.8% after being coated with PDMS@STA-KL. Cone calorimeter test results showed that the peak heat release rate and the total heat release of lignocellulose composites coated with PDMS@STA-KL decreased by 18.7% and 19.2% from those of uncoated lignocellulose composites, respectively. This multifunctional lignocellulose composite surface with improved water repellency and flame retardancy could open new avenues in the field of novel lignocellulose-based materials.

## Figures and Tables

**Table 1:** Cone calorimeter data of LC and LC coated with PDMS@STA-KL.

Samples	TTI ^a^ (s)	PHRR ^a^ (kW/m^2^)	TPHRR ^a^ (s)	FIGRA ^a^ (kW/m^2^ s)	THR ^a^ (MJ/m^2^)
LC	33	295.5	256	1.2	65.2
Coated LC	73	240.2	284	0.8	52.7

^a^ TTI, PHRR, TPHRR, FIGRA and THR refer to the time to ignition, peak heat release rate, the time from ignition to achieve PHRR, fire growth rate (acquired through dividing PHRR by TPHRR), total heat release, respectively.
